# Pain after osteoporotic fractures using mouse models and patient samples

**DOI:** 10.1007/s00774-025-01677-w

**Published:** 2025-12-25

**Authors:** A. Radulescu, M. Hopkinson, Y. Chang, A. Azfer, S. Ralston, C. Chenu

**Affiliations:** 1 Department of Comparative Biomedical Sciences, Royal Veterinary College, 4 Royal College Street, London, NW1 OTU UK; 2 Centre for Genomic and Experimental Medicine, Institute of Genetics and Cancer, University of Edinburgh, Western General Hospital, Edinburgh, EH4 2XU UK

**Keywords:** Osteoporosis, Fractures, Pain behaviours, Pain genes

## Abstract

**Introduction:**

Osteoporosis can cause chronic pain, but the mechanisms are unclear. This study investigates pain behaviours in mouse models of osteoporosis and fracture together with nociceptive markers expression in bone and dorsal root ganglia (DRGs). It also quantifies nerve markers in serum of patients with or without osteoporotic fractures and pain.

**Material and methods:**

Ovariectomy (OVX) or Sham surgery (Sham-OVX) of C57/Bl6 mice was performed (*n* = 10/group) and evoked and spontaneous pain behaviours assessed. In another experiment, OVX or Sham-OVX mice underwent a femoral osteotomy or sham osteotomy (*n* = 8/group) and pain behaviours measured. Gene expression of pain markers in bone and DRGs was quantified by RT-PCR. Nerve markers were quantified in serum of osteoporotic patients with or without fractures and pain using specific ELISAs.

**Results:**

OVX did not cause changes in pain behaviours nor alter nociceptive gene expression in bone and DRGs. Osteotomy and Sham osteotomy both affected pain behaviours in mice compared to non-operated controls but did not significantly change nociceptive gene expression in bone and DRGs. OVX before osteotomy worsens weight-bearing compared to Sham-OVX. Fracture and pain did not affect nerve markers expression levels in serum of osteoporotic patients.

**Conclusion:**

This study demonstrates that OVX and subsequent bone loss in mice are insufficient to induce pain behaviours but may intensify pain after fracture. Our clinical analysis does not show a correlation between circulating nerve markers and fracture pain reported by the patients but suggests possible sex differences in pain markers that need to be further investigated.

**Supplementary Information:**

The online version contains supplementary material available at 10.1007/s00774-025-01677-w.

## Introduction

Osteoporosis is the main cause of fragility fractures which represent a massive public health burden [1, 2]. It is usually silent in 70% of the cases, which leads to fractures going undetected and underdiagnosed [3]. Bone pain is the osteoporotic women’s biggest complaint, particularly if associated with undetected vertebral fractures [4, 5], vertebral osteoporosis, deformities and back pain [6–9]. It is essential to identify women with undetected fractures and manage persistent pain [10–12].

Bone is highly innervated by nerve fibres involved in skeletal development, maintenance and repair [13–15]. The distribution of sensory nerve fibres in bone, their neuronal sub-types and bone pain mechanisms have been well described [16–18]. Osteoporosis is a multifactorial systemic disease and pain measurements in this condition are rare. Deep musculoskeletal pain is frequent and the causes range from increased bone resorption [19], nerve sprouting [20], but also changes in oestrogen levels, mechanical loading and undetected fractures [21].

Animal models are useful to study pain, particularly rodents [22]. Ovariectomy (OVX) mimics postmenopausal osteoporotic bone loss [23] but reflects less dramatic loss of oestrogens after menopause [24]. It is now used as a model to investigate osteoporotic pain, requiring methods that quantify “pain-like behaviours” via testing responses to an external stimulus or measuring spontaneous behaviours [25]. Many are indirect measures of pain as they do not involve the direct stimulation of nociceptive fibres in bone [26].

The measurement of circulating biomarkers of pain increasingly supports the diagnosis and efficacy of therapies in skeletal disorders. Neurotrophins are important for the ingrowth and maintenance of nerve fibres in bone [27]. Their levels in serum, such as Nerve Growth Factor (NGF) and Brain-Derived Neurotrophic Factor (BDNF), have previously been linked with disease activity and pain in rheumatoid arthritis, bone cancer pain and bone injury [28, 29]. Similarly, the expression of the neurotransmitter Calcitonin Gene-Related Peptide (CGRP) in serum is related to osteoarthritis severity and pain [30].

Our study examined whether OVX-induced bone loss in mice is painful before fracture onset and correlated pain behaviours in these mice with transcriptional changes in pain-related genes. We also determined whether osteotomy after OVX is more painful than osteotomy in control mice. Finally, we performed a small clinical study to quantify CGRP, NGF and BDNF in serum of patients with and without fractures and reported pain.

## Material and methods

### Animals studies

All animal procedures were performed under the Animal (Scientific Procedures) Act 1986 and the Royal Veterinary Colleges’ Animal Welfare and Ethical Review Body under the Project License 70/8782. ARRIVE (Animal Research: Reporting of In Vivo Experiments) guidelines were followed for study designs.

### Study design

Young and mature adult female adult C57BL/6 mice (11–12 weeks old or 30 weeks old at surgery) were acclimatised to the BSU (Biological Services Unit) for at least one week before undertaking any procedure and kept on a 12-h (7:00/19:00) light/dark cycle with food (Rodent Diet 20 5053, LabDiet, PMI Nutrition International, Missouri, USA) and water ad libitum. Two age groups of mice were used to investigate the effect of ageing on pain behaviours and osteoporosis-related bone loss.

### Ovariectomy

To model postmenopausal osteoporosis, bilateral ovariectomy (OVX) or Sham surgery (Sham) of young and mature adult female C57/Bl6 mice was performed (*n* = 10/group). In the study with mature adult mice, a non-operated non-anaesthetised control group was added to account for the possible pain induced by the Sham procedure which involves the same surgery method except that clamping and cautery of the ovaries were not performed. Bilateral ovariectomy was performed as previously described [31]. Pain behaviours were assessed at baseline (before OVX) and weekly from weeks 1 to 6 after OVX using evoked (hot (50 °C)/cold (15 °C) plate, Von Frey) and naturalistic behaviours (burrowing and nesting).

### Fracture surgery

Mice were OVX or had Sham surgery when 6 weeks old and underwent a unilateral femoral osteotomy maintained by an external fixator (ExFix) six weeks after OVX. These two groups refer to OVX-ExFix and Sham-OVX-ExFix, respectively. Another group was a sham osteotomy one (OVX-ShamExFix). All groups had *n* = 8 mice. Femoral osteotomy was previously performed and the source of fixator, the procedure for Sham osteotomy, pre- and post-operative care and pain management were described before [32]. Briefly, mice were induced with isoflurane and administered analgesic subcutaneously: buprenorphine (0.1 mg/kg) and Meloxicam (20 mg/kg). A 13 mm incision was made on the right thigh and a blunt dissection performed to visualise the femur. Once the femur was exposed, a spatula was inserted below the femur to stabilise the bone and protect the surrounding tissue at the drilling site. Four titanium screws were screwed into the bone through an external fixator (Mouse ExFix, RISystem). 0.33 mm holes were drilled for the fixator pins to be then screwed in, ensuring that the fixator is stabilised and the pins are parallel to each other. For the Sham procedure, the surgery stopped here and continued with the closing-up step onwards. For the ExFix osteotomy procedure, an osteotomy was performed with the giggly wire, which was carefully slid under the femur and clipped on both ends with haemostats that allowed a clean cut of the bone mid-shaft by the friction of the Gigli wire against the bone. The Gigli wire was removed once the osteotomy was completed, leaving a 0.25 mm fracture gap. The femoral muscle was closed using a single interrupted suture and the incision closed with Vycril 5–0 reverse cutting PC-1. Animals were singly housed overnight for the first three days post-operatively. Meloxicam and buprenorphine were administered subcutaneously every 24 h for the first three days or more when pain scores indicated their need. Animals were weighed daily for seven days following the surgery and pain behaviours assessed weekly from 7 days post-surgery (during which no behaviours tests were performed to avoid the effects of analgesics) to 6 weeks after fracture surgery.

### Behaviours measurements

Before the weekly testing habituation, training and baseline sessions were performed to allow for acclimatisation to the equipment and to eliminate fear/ unfamiliarity bias from the response. Researchers performing behavioural testing were blinded to the conditions of the mice.

#### Non-evoked behaviours

Nesting building and burrowing activity were assessed for 2 h or overnight at the same time. Nesting building was scored on a scale from 1 to 5 according to a visual assessment of the degree of complexity of the built nest during the designated period [33]. Mice were provided with 200 g filled burrowing tubes, built according to a previously published design [33]. The amount burrowed was recorded by subtracting the amount left in the tube at the end of the testing timeslot from the 200 g of food pellets. Each animal was assigned randomly to one burrowing tube, kept the same throughout the study. Another non-evoked behaviour measured in the fracture study was static weight bearing in mice (Linton instrumentation, UK). It was recorded for each limb three times once the mouse had stabilised in the restraining cup and had distributed its body equally on both plates.

#### Evoked behaviours

Mechanical allodynia was measured by applying graduated levels of Von Frey filaments (Aesthesio Precision Tactile Sensory Evaluator, DanMic, California, USA) to the left hind paw for 2 seconds, ten times at each hair weight, as previously described [34]. The withdrawal threshold was determined as five out of 10 positive responses, being defined as a fast withdrawal of the paw. Results are expressed as threshold force (g) at which 50% of the responses are of withdrawal (5 positive hits out of 10 tested for each filament in an incremental manner of force). Thermal sensitivity is assessed by plantar exposure to a hot or cold plate. Hot/ Cold plate latency was recorded as the number of seconds passed since laying the mouse on the plate until the mouse showed withdrawal signs, such as vocalisation and paw licking. The results are expressed in seconds without the mouse’s withdrawal signal.

### Processing of biological samples

Six weeks after fracture surgery or OVX, animals were sacrificed by CO2, followed by confirmation of death by heart puncture (exsanguination). Blood samples were obtained from mice by cardiac puncture and serum samples aliquoted and stored at − 80 °C. Lower limbs and lumbar dorsal root ganglia (DRGs) were dissected; DRGs were flash-frozen in dry ice once isolated whilst femurs were collected from both limbs, bone marrow flushed and RNA extracted from bone.

### q-RT-PCR analysis

Gene expression profiles of pain markers (panel of neuropeptides, neurotrophins and ionic channels) in bone and DRGs were measured by quantitative RT-PCR (RT-qPCR) using TaqMan Assay technology. Results are expressed as fold change between OVX and Sham and Fracture compared to Sham-Fracture using the Δ ΔCt method. For statistical comparisons, a two-way ANOVA was performed and the ΔCts of the control and the test conditions were compared. Significance was indicated as *p* value < 0.05. The primers used are shown in Table 1. Briefly, bone and DRGs were separately crushed with a 0.5 mm stainless steel bead in a tube with Qiazol RNA extraction buffer using the Tissue Lyser II (Qiagen, Maryland, USA). The lysate was transferred to 1.5 ml micro-centrifuge tubes for further RNA extraction using the commercial ReliaPrepTM miRNA Tissue Miniprep (Promega, USA) kit. RNA integrity and quantity were measured using DeNovix (Wilmington, USA) fluorometric quantification equipment. cDNA synthesis was performed using the SuperScriptTM IV VILOTM Master Mix (Thermo Fisher, Waltham, Massachusetts, US). The cDNA reactions were then used for TaqMan assays. We followed as much as possible the MIQE guidelines [35]. Data analysis of the qPCR data was performed as follows: average Ct was computed from the three technical replicates. Data were normalised to the Ct of the housekeeping gene GAPDH (glyceraldehyde 3-phosphate dehydrogenase). TaqMan assay has an internal probe control of GAPDH, so the DCt was computed by subtracting the corresponding internal housekeeper control equivalent to GAPDH from the Ct of the gene of interest in the respective well. For the OVX study, DDCt was calculated by subtracting the average Sham DCt from the OVX DCt to give the relative expression of the genes in the OVX condition to the Sham condition. The fold change 2-DDCt was then calculated and a bar graph plotted as M ± SEM. For the OVX effect in ExFix osteotomy mice, DDCt was calculated by subtracting the average ShamOVX-ExFix DCt from the OVX-ExFix DCt average to give the relative expression. For the ExFix effect in OVX mice, DDCt was computed by subtracting the average OVXShamExFix DCt from the OVX-ExFix DCt average to get the relative expression.

**Table 1:**
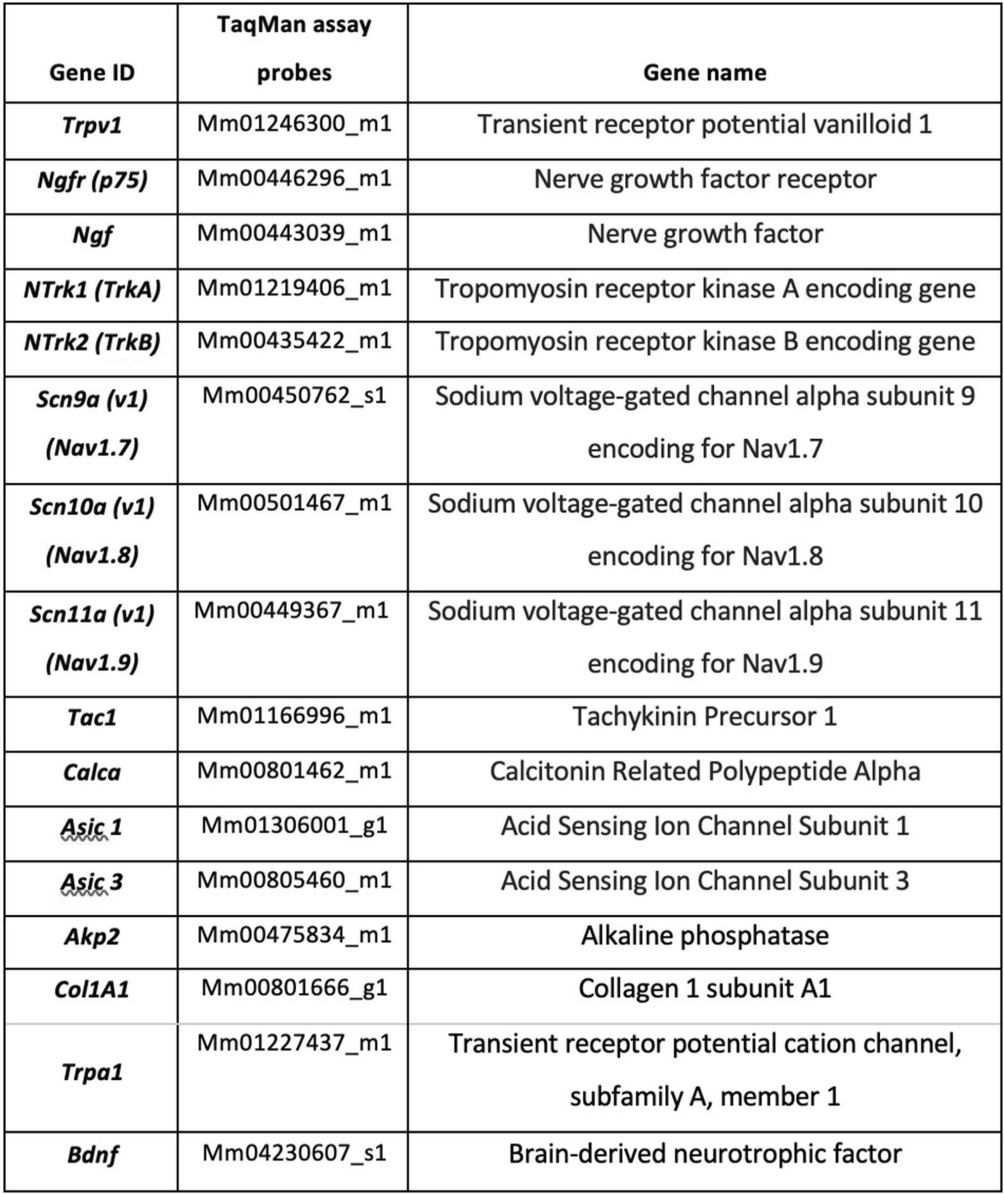
TaqMan Assay probes

### Human studies

Serum samples were kindly given by Prof Stuart Ralston, Edinburgh, UK. They were part of another study (Genetics of Bone and joint Disease, REC reference 04/S1102/41) approved by Lothian NHS board. All patients gave their informed consent. This study was approved by the Clinical Research Ethical Review Board of the Royal Veterinary College. Serum was previously collected from patients ranging from 30 to 90 years old from both males (*n* = 35) with (*n* = 18) or without (*n* = 17) fracture and females (*n* = 58) with (*n* = 30) or without (*n* = 28) fracture. Age refers to age at the time of blood sampling. All patients with fracture had pain reported by the patient, whilst patients with no fracture had no pain. In females, age ranged from 40 to 90 years, and in males from 30 to 80 years. Patients were grouped into three age groups: under 50, between 60–69 and over 70 years old. Most samples were from osteoporotic patients, 58/62 in females (95%) and 35/41 in males (85%). Only the osteoporotic samples were included in this study. The time between pain reports at DEXA scan timepoint and blood sampling was variable within the sample pool, averaging 130. 52 ± 74.24 days (mean ± SEM). Patients with conflicting comorbidities were not chosen in this study.

### ELISAs for nerve markers in clinical serum samples

Serum from osteoporotic fracture patients was stored at − 80 °C. Neurotrophins (NGF and BDNF) and neuropeptide CGRP were quantified in serum of osteoporotic patients with or without fractures using specific ELISAs. The following kits were used for the detection of NGF (Human beta-NGF DuoSet ELISA, # DY256, R&D, UK), CGRP (Human CGRP ELISA Kit, # CSB-E08210H, CUSABIO, US) and BDNF (Total BDNF Quantikine ELISA Kit, #DBNT00, R&D Systems, UK). Three plates of each kit were used with a standard curve on each, which were comparable between plates. The distribution of samples on each plate was done at random and the user was blinded to the samples. For the NGF kit, the majority of male samples were out of the detection limits. Hence, only female data were considered where the sample size (*n* = 8) was comparable between both conditions. For the BDNF and CGRP kits, there were *n* = 18 males with fractures, *n* = 17 males without fractures, *n* = 30 females with fractures, and *n* = 28 females without fractures.

### Statistics

Analysis was performed using Prism Version 10.0.3 except for the human fracture study where it was performed using IBM SPSS version 28.0.0.0. Descriptive statistics and Normality tests were performed on each data set and all data are expressed and represented as Mean ± Standard Error of the Mean (M ± SEM). Two-way repeated measures ANOVA (or mixed effects model if there was missing data) and Dunnett’s post hoc test were employed to capture differences between groups for qPCR, weights and behaviours and for changes in different time points. For the human vertebral fracture analysis of sex and age inferences, a general linear model was fitted with plate number as covariate, age group and sex, fracture status and an analyte serum concentration as factor. Mean analyte concentrations are shown as M ± SD. For ELISA measurements, results were interpolated using a 4-Parameter Logistics (4-PL) curve and multiplied by the dilution factor to get the concentration of the unknowns. Two-sample test was used to compare the mean levels of analytes in serum between OVX and Sham, and between fracture groups.

## Results

### Ovariectomy doesn’t affect pain-like behaviours compared to Sham surgery but surgery pain is present

**Fig. 1:**
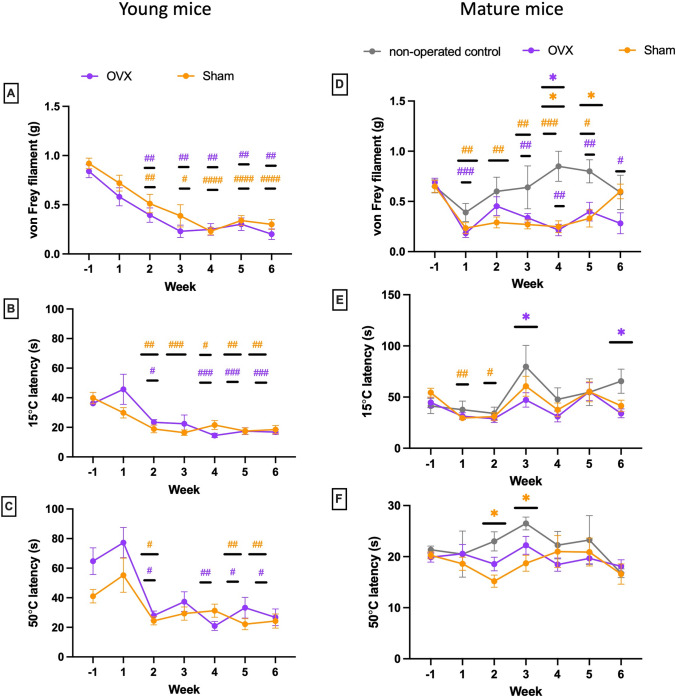
Effect of ovariectomy on pain-like behaviours. Evoked behaviours were assessed in young (11–12 weeks old) OVX and Sham-operated mice (*n* = 10 per group) as well as in mature adult OVX (30–32 weeks old, *n* = 9) mice, Sham-operated mice (*n* = 10) and non-operated controls (*n* = 4). Evoked pain-related behaviours were measured weekly from one week before surgery (week-1, baseline) to week 6 after surgery. Young mice: **A** mechanical allodynia (withdrawal threshold), **B** thermal allodynia (cold plate latency at 15 °C). **C** Thermal hyperalgesia (hot plate latency at 50 °C. Mature adult mice: **D** mechanical allodynia (withdrawal threshold), **E** thermal allodynia (cold plate latency at 15 °C). **F** Thermal hyperalgesia (hot plate latency at 50 °C). Results are expressed as M ± SEM and are normally distributed. Two-way repeated measures ANOVA, Dunnett’s multiple comparisons tests for differences within each group: ^#^(p ≤ 0.05), ^##^(p ≤ 0.01), ^###^(p ≤ 0.001), ^####^(p ≤ 0.0001) compared to baseline OVX and Sham. *(p ≤ 0.05) compared to non-operated controls. Tukey’s multiple comparisons test for group differences: no significance (ns)

OVX in young (11 and 12 weeks old) or mature adult (30–32 weeks old) mice did not cause significant changes in evoked pain behaviours (Fig. 1). In young mice, a statistically significant decreased threshold in mechanical sensitivity was established for both Sham and OVX groups 2 weeks after surgery in comparison to their respective baselines that was maintained at 6 weeks post-surgery (Fig. 1A). There was, however, no difference between Sham and OVX groups at any of the timepoints tested. Similarly, Latencies to cold (Fig. 1B) and heat (Fig. 1C) sensitivities decreased post-surgery in both the OVX and sham groups in all weeks and did not recover when compared to baselines during the 6-week follow-up post-surgery. In mature mice, the thresholds to mechanical sensitivity in OVX and Sham mice were significantly lower than non-operated control mice (Fig. 1D), indicating that OVX and Sham surgeries increased mechanical sensitivity. Sham-operated mice recovered to levels comparable to the non-operated group six weeks after surgery but OVX mice did not. OVX surgery did not cause sensitivities to heat and cold in mature mice (Fig. 1E, F).

Spontaneous behaviours, nesting and borrowing assessed overnight, were not affected by OVX (data not shown).

### Nociception-related genes are differentially expressed in bone and DRGs but are not significantly regulated by ovariectomy

**Fig. 2:**
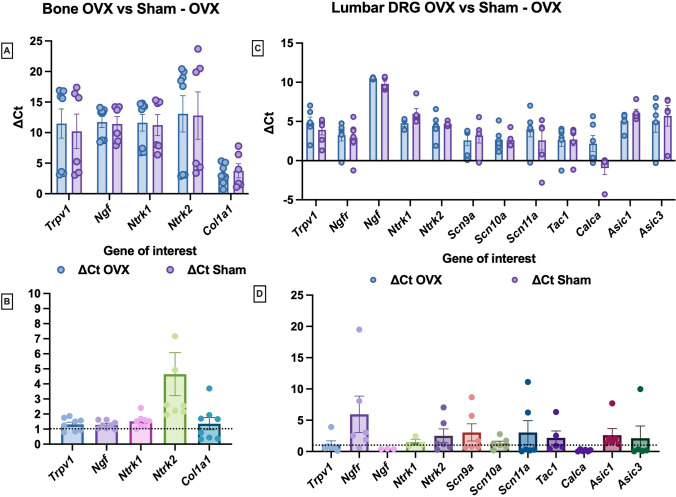
Effect of ovariectomy on nociceptive genes expression in bone and dorsal root ganglia. Femoral bone and lumbar DRGs corresponding to vertebrae L3–L6 were dissected from 30 to 32-week-old C57BL/6 female mice that were OVX (*n* = 6) or had Sham surgery (*n* = 6) and used for TaqMan assays. Pain gene panel expression was quantified in bone (**A, B**) and lumbar DRGs (**C, D**) of OVX mice relative to Sham mice. **A, C** ΔCt of OVX and Sham for each gene of interest for femoral bone or lumbar DRGs. **B, D** Relative gene expression in bone and Lumbar DRGs shown as fold change expression in OVX mice relative to Sham mice used as controls. Results are expressed as M ± SEM. Two-Way ANOVA show no statistical differences

Expression of genes important in pain pathways was analysed in bone and lumbar DRGs from Sham- and OVX-old mice 6 weeks after surgery (Fig. 2). Lumbar DRGs were chosen as they transmit sensory information from the lower limbs to the central nervous system. Twelve nociceptive and neurotrophic genes were chosen in our panel. Only a few nociception-related genes in the panel were detectable in all bone samples (Fig. 2A), in contrast to lumbar DRGs where all the genes in the panel were expressed (Fig. 2C). In bone, *NTRK2* coding for tropomyosin receptor kinase B, a receptor for BDNF, was the only significantly upregulated gene in the OVX group compared to Sham (Fig. 2B). In lumbar DRGs, some genes were upregulated in the OVX group, but their expression levels did not change significantly compared with the Sham control group (Fig. 2D).

### Fracture affects pain behaviours compared to Sham Fracture and ovariectomy before fracture surgery can worsen pain

**Fig. 3:**
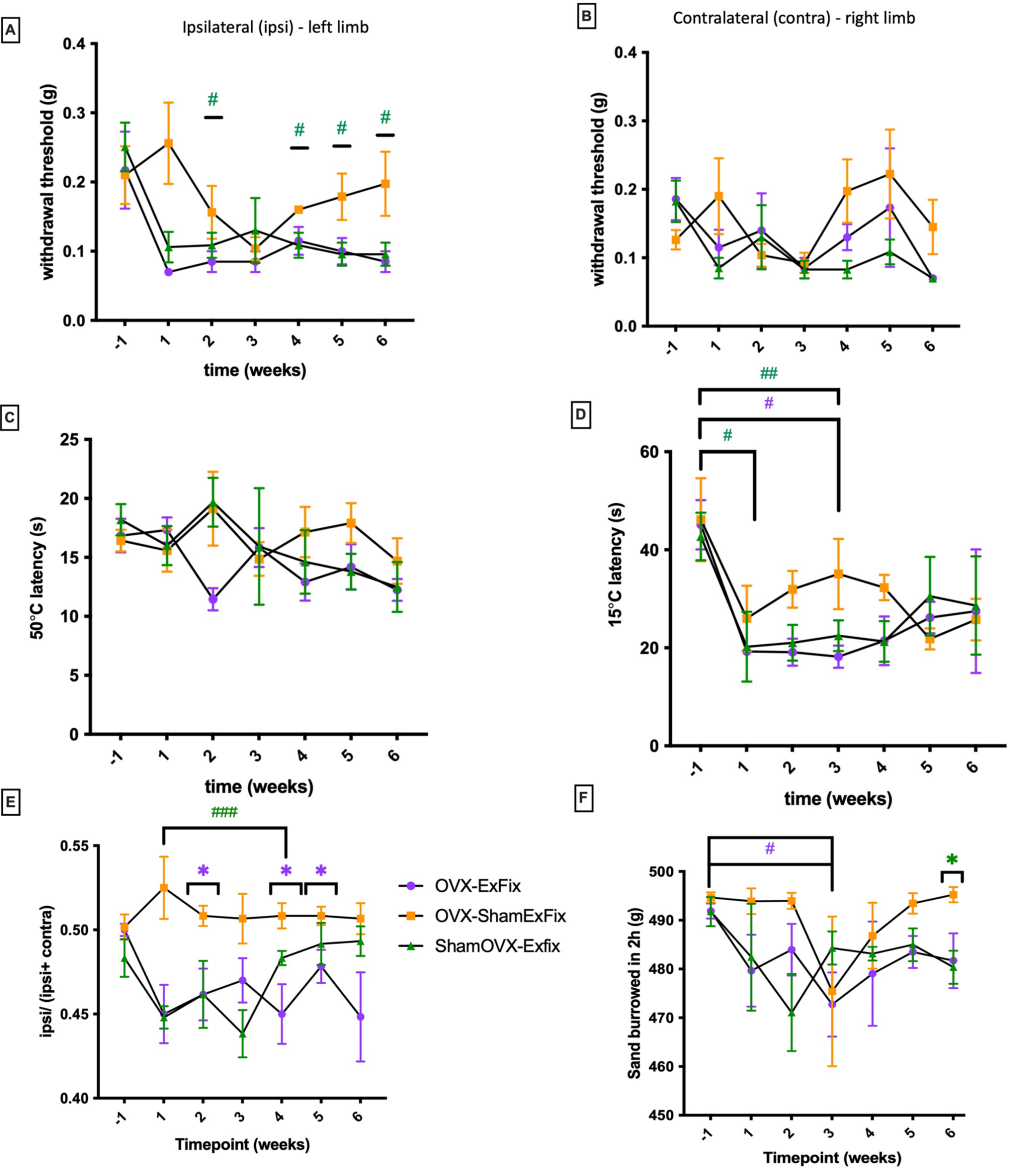
Effect of ovariectomy and fracture on pain-like behaviours. Mice were OVX or had Sham surgery when 6 weeks old. At 12 weeks old, OVX or Sham mice underwent a unilateral femoral osteotomy (OVX-ExFix and ShamOVX-ExFix, respectively) or Sham osteotomy (OVX-ShamExFix) (*n* = 8/group). Evoked and spontaneous behaviours were assessed in mice from week 1 (before fracture surgery) to week 6 endpoint. **A** Mechanical allodynia (withdrawal threshold) on **A** ipsilateral-left operated leg and **B** contralateral-right control leg. **C** Thermal hyperalgesia (hot plate latency at 50 °C). **D** Thermal allodynia (cold plate latency at 15 °C). **E** Weight-Bearing (WB) shown as the ratio of weight bearing on the ipsilateral to the combined weight bearing on both ipsi- and contra-lateral legs. **F** Burrowing measured as sand burrowed out of 500 g during a 2-h window (food and water were provided ad libitum). Two-Way ANOVA and significance shown in comparison with OVX-ShamExFix as *(p ≤ 0.5) and in between time points and baselines as ^#^(p ≤ 0.05), ^##^(p ≤ 0.01) and ^###^(p ≤ 0.001)

We next analysed if pain behaviours were changed after fracture in OVX mice. Evoked and spontaneous behaviours were analysed weekly during 6 weeks after osteotomy (Fig. 3). The withdrawal threshold in the operated leg in both osteotomy groups (OVX-ExFix or Sham OVX-ExFix) dropped at week 1 post-surgery in comparison with the pre-surgery baseline (Fig. 3A). This was constantly lowered during the 6 weeks period. In contrast, the ShamExFix group had a significant recovery at week 6 in comparison to both osteotomy groups. A higher threshold for mechanical sensitivity was observed in the OVX-ExFix group compared to the Sham OVX-ExFix group in the first three weeks after surgery, close to significance (Fig. 3A). There was no difference between time points or conditions in the contralateral leg (Fig. 3B). The threshold for heat sensitivity showed an overall decreasing trend in all three surgical groups with no differences between groups (Fig. 3C). The cold sensitivity threshold was decreased in all groups, more severely in the osteotomy ones (Fig. 3D), but this was improved gradually and at the end point the cold sensitivity was similar for all groups. Weight-bearing, commonly used as a spontaneous measure of pain, was assessed in the operated leg relative to the total weight borne on both legs. Both osteotomy groups had a consistently decreased weight bearing on the operated leg in the post-operative weeks (Fig. 3E). This lasted for all 6 weeks post-operatively for the OVX group, whilst the ShamOVX group recovered to pre-operative levels from week 4 onwards. This suggests an exacerbation of pain and disability in the OVX group. The OVX-ShamExFix group did not exhibit a weight-bearing deficit during all 6 weeks post-operatively (Fig. 3E). Burrowing was very variable in this study. Six weeks after fracture, only the Sham-ExFix group has recovered the burrowing capacity to the same level as the pre-operative baseline, indicating that osteotomy negatively impacts burrowing regardless of whether mice had a ShamOVX or an OVX (Fig. 3F).

### Expression of nociception-related genes in bone and DRGs are not significantly changed by fracture surgery in Sham and ovariectomised mice

**Fig. 4:**
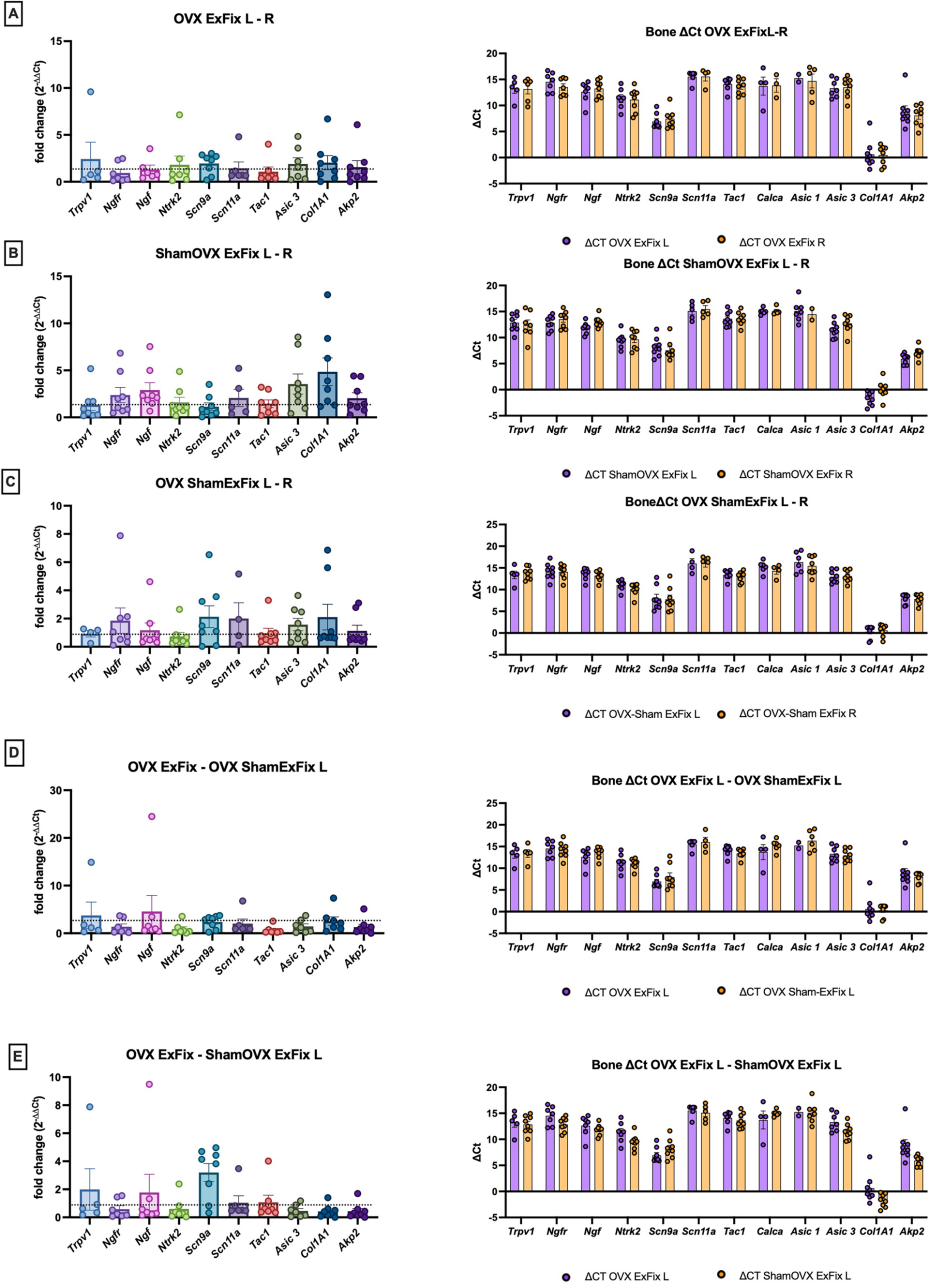
Effect of ovariectomy and fracture on nociceptive genes expression in bone. Pain gene panel expression was quantified in femoral bone in the OVX ExFix mouse model and illustrated both as relative fold change (left panels) and ∆Ct (right panels). The first comparison was made between operated versus control limb. Results are shown in the ipsilateral-operated left limb (L) in comparison to the unoperated control limb contralateral right (R) in **A** OVXExFix (*n* = 8), **B** ShamOVXExFix (*n* = 8) (**B**) and **C** OVX ShamExFix (*n* = 8). The second comparisons were made between OVXExFix and OVX ShamExFix in the operated L limb (**D**) and between OVXExFix and ShamOVXExFix in the operated L limb (**E**). Bar graphs show individual values M ± SEM. Statistics: Sample t tests between ∆Ct of L and R revealed no significance between values

Relative nociception-related gene expression in femoral bone was quantified by qPCR. For each surgery group (OVX-ExFix, ShamOVX-ExFix and OVX-ShamExFix), the relative expression in the left operated limb to the right contralateral limb was calculated and indicated as L-R (Fig. 4A–C). Most nociception-related genes were upregulated in femoral bone of the fracture limb compared to the non-fractured one, but the changes were only of 1.5- to 2-fold (Fig. 4A–C). The genes most affected in the fracture groups were Trpv1, Scn9a, Asic3 and CollA1. When comparing gene expression in bone from the fractured limb between the OVX ExFix group and the OVX Sham-ExFix group, a few genes were upregulated including Trpv1 and Ngf, whilst Ngfr and Tac-1 were down-regulated (Fig. 4.D). OVX before fracture led to an increase in expression of TrpV1, Ngf and Scn9a in the fracture limb (Fig. 4E).

**Fig. 5:**
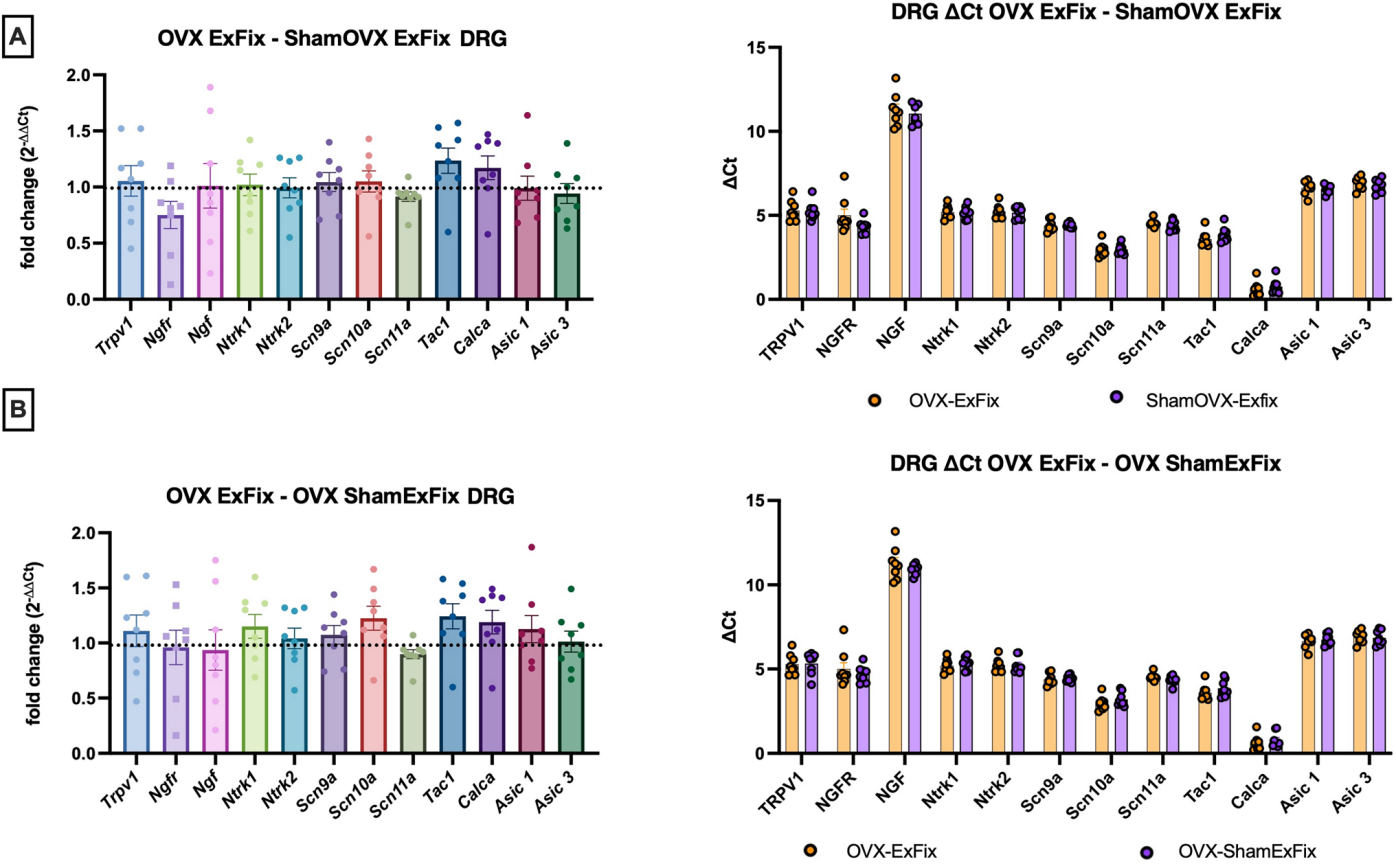
Effect of ovariectomy and fracture on nociceptive genes expression in DRGs. Pain gene panel expression was quantified in DRGs in the OVX ExFix mouse model and shown both as relative fold change (left panels) and ∆Ct (right panels). **A** Relative gene expression in DRGs shown as fold change and ∆Ct of pain gene panel for Lumbar DRGs OVXExFix (*n* = 8) to ShamOVXExFix (*n* = 8) and **B** OVXExFix to OVX ShamExFix (*n* = 8). Bar graphs show individual values M ± SEM. Statistics: Sample t tests showed no significance between ∆Ct for each comparison

All genes were highly detectable in DRGs samples. No effect of osteotomy was observed in nociception-related gene expression in DRGs when comparing expression in the OVX-ExFix group to the OVX-ShamExFix (Fig. 5B). Similarly, nociception-related genes expression in DRGs was similar in the OVX-ExFix and the ShamOVX-ExFix groups (Fig. 5A).

### Circulating neuronal biomarkers do not correlate with fracture pain in osteoporotic patients

**Fig. 6:**
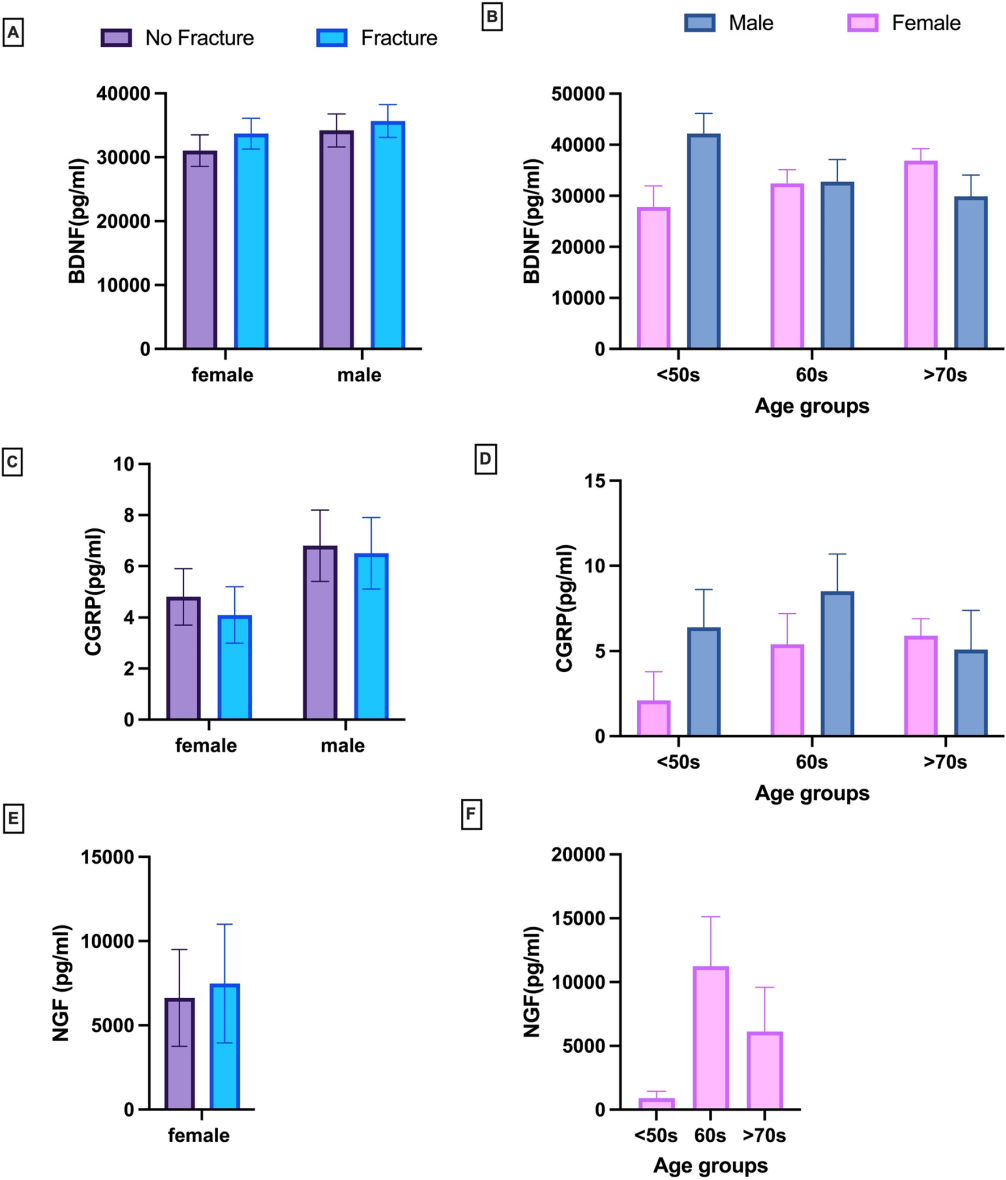
Quantification of circulating nerve markers in osteoporotic patients. Brain-Derived Nerve Factor (BDNF), Calcitonin Gene-Related Peptide (CGRP), and Nerve Growth Factor (NGF) were measured in serum of male and female osteoporotic patients with or without fracture and pain using specific ELISAs. Results are shown in relation to fracture and age. **A** BDNF levels related to Fracture; **B** BDNF levels according to age; **C** CGRP levels related to fracture; **D** CGRP levels according to age; **E** NGF levels related to fracture; **F** NGF levels according to age. Bars show M ± SD. Two-way ANOVA was performed to detect differences between male and female and fracture versus no fractures within groups. Statistical significance: NS

Nerve markers were quantified by ELISA in serum samples from patients with or without fracture and pain. The expression was analysed per sex, shown as males (M) and females (F) and by age groups (below 50 s, 60 s and above 70 s). The age grouping was well balanced. BDNF expression levels in serum were not significantly changed in males and females with or without fracture (Fig. 6A). The male group of under 50 s had a higher level of serum BDNF in comparison with the over 70 s group but this wasn’t significant (Fig. 6B). CGRP was not significantly changed in males or females with or without fracture (Fig. 6C). There were no significant differences amongst the age groups (Fig. 6D). NGF was not detected in most male serum samples and therefore only the data for female are shown. There was no significant change in NGF expression in female between Fracture and no Fracture (Fig. 6E). The levels of NGF were lower in the under 50 s group when compared to the 60 s and 70 s age groups, but not significantly (Fig. 6F). These results demonstrate that it is possible to quantify these markers in serum and that their expressions mostly increase with age but do not correlate with fracture pain.

## Discussion

This experimental study performed in female mice showed no changes in evoked and naturalistic pain behaviours after OVX in young and mature mice, although surgery pain was present in both Sham and OVX groups until 6 weeks after surgery. Micro-CT analysis of bones 6 weeks after OVX (Figure S1, supplementary material) confirmed the bone loss after OVX and the validity of our model. This suggests that OVX and its resulting bone loss do not induce pain behaviours in mice, recapitulating the clinical non-painful presentation of some osteoporotic patients [9]. This data contrasts with other studies using similar models in rodents, where OVX mice had lower pain thresholds on Von Frey tests 10 weeks after surgery [36] and plantar hypersensitivity to mechanical, cold and heat stimuli 8 weeks after surgery compared to Sham mice [26]. Our work didn’t examine persistent pain for many weeks after surgery and this can be a limitation considering the slow onset of pain and the long duration of hyperalgesia in osteoporosis. Pain hypersensitivity was maintained for 6 months in OVX mice in Susiki’s study [26]. Nevertheless, our demonstration that in mature mice the sham and OVX mice had reduced mechanical sensitivity thresholds compared to non-operated controls and that Sham mice recovered to levels comparable to the non-operated group after 6 weeks but not the OVX mice suggests that the surgical trauma induces more severe mechanical hypersensitivity in the OVX group. Mechanically induced sensitisation was similarly previously reported after OVX in mice [37]. We didn’t show any consistent effect of OVX on cold or hot sensitivity in either age group or surgery group for 6 weeks after surgery. This contrasts to previously described enhanced sensitivity to thermal stimuli after OVX in mice [38] and could be explained by the methods employed to quantify pain behaviours in rodents. As there is no direct stimulation of bone nociceptive nerve fibres with the evoked pain behaviours used in animal studies, the pain measured is indirect [22]. Several studies have shown that pain assessed after OVX is mostly a cutaneous pain hypersensitivity [26] or a visceral pain [38]. Deep musculoskeletal pain is poorly assessed after OVX, nor spontaneous behaviours which better mimic the pain experienced by osteoporotic patients. The present study did not show changes in burrowing and nesting overnight after OVX, may be due to an increased familiarity with the procedure with time. Comparing pain behaviours in young and mature mice, we couldn’t demonstrate that ageing mice more frequently presented pain-related behaviours.

This study directly evaluates changes in expression of a pain gene panel in bone and DRGs after OVX. Neurotrophins, neuropeptides and nociceptive genes were included in this panel. To our knowledge, few studies have previously examined nociceptive gene expression in bone. Nociceptors, such as Transient Receptor Potential Vanilloid type 1 (TRPV1) and Acid-Sensing Ion Channels (ASICs), are expressed in bone tissue and the expression of ASIC 1 and 2 are significantly increased by OVX [39]. NGF is also expressed in bone during fracture repair [40]. Our work shows that few nociceptive genes are expressed in bone compared to DRGs. It confirms that genes coding for NGF and its receptors are expressed in bone. Similarly, *NTRK2* coding for tropomyosin receptor kinase B (TrkB), a receptor for BDNF was identified in bone. Both neurotrophins are known to affect bone metabolism [40]. All the nociception-related genes analysed were expressed in DRGs. It is known that the majority of DRG neurons innervating the bone contain CGRP, BDNF and tyrosine receptor kinase A (TrkA) [40, 41]. However, this study indicates that nociceptive gene expression in mouse DRGs was not significantly affected by OVX, which is in line with our behaviours data. Although most genes were upregulated by OVX, their expression levels didn’t change significantly. A previous study has shown that CGRP is increased in rat trigeminal ganglions after OVX [42].

Clinical data show both acute and chronic pain after fractures [43]. We previously reported using the same model of osteotomy in mice high levels of invoked nociception after fracture [32]. We discussed in this study [32] that this diaphyseal fracture model with external fixation doesn’t represent the best pre-clinical model to study osteoporotic fractures, but it provides increased stability, a requirement for fracture healing, and is highly standardised which makes this model highly relevant for studies examining pain behaviours. Our study confirms that mechanical hypersensitivity was increased after osteotomy (ExFix) compared to Sham surgery but not thermal sensitivity. The development of mechanical hypersensitivity was accompanied by altered weight-bearing. The comparisons between the Sham-OVX and the OVX groups showed no significant differences for mechanical and thermal measurements after osteotomy. In contrast, we show that OVX before osteotomy worsens the weight bearing deficit in the operated leg compared to Sham-OVX. Weight-bearing is frequently used to evaluate post-surgical, neuropathic and inflammatory pain. Our results suggest that whilst OVX alone does not affect pain behaviours, it may intensify pain after osteotomy. OVX influences fracture repair [44, 45], suggesting that inflammation, vascularisation and nerve sprouting could be affected by OVX, affecting pain behaviours. In a clinical scenario, bone repair is also delayed in osteoporosis and the pain is prolonged becoming chronic and neuropathic [46]. Our study shows no consistent significant changes in nociceptive gene expression after fracture in bones and DRGs. This could be due to our model of osteotomy with rigid fixation where less inflammatory response is triggered after fracture, but also to the fact that we measured gene expression only at one time point after fracture. This is a limitation of our study which does not allow to capture fluctuations in nociceptive gene expression at different stages of fracture repair nor the different dynamics of pain.

Changes in neuronal circulatory markers may be associated with pathological conditions and pain. We chose to quantify NGF, BDNF and CGRP in serum of osteoporotic patients due to their contribution to pain signalling and involvement in bone metabolism and diseases [28, 30, 47]. To our knowledge, this is the first clinical study trying to correlate the expression of circulating biomarkers with pain in osteoporotic patients. Our small study using 100 serum samples from patients with or without osteoporotic fractures and reported pain showed that CGRP, BDNF and NGF were detected in serum of osteoporotic patients and all expressed within the detection limits of the assays. However, our data demonstrate that fractures and pain did not affect their expression levels in serum of male and female patients. This does not support the use of these nociceptive factors as circulating biological markers of osteoporotic fracture pain. One limitation to this study is the fact that the serums were collected at different times after fracture occurrence, whilst pain is a dynamic process during fracture repair. One interesting result is that NGF levels in most male samples were undetectable as opposed to females indicating a possible sex difference in the circulating levels of NGF. NGF protein expression is regulated by oestrogen in ovariectomised mice [48], suggesting gender differences in pain sensation. Our data also indicate that both NGF and CGRP serum levels are increased in female with the age of the patients. This hasn’t been previously reported and the causal role of this increase and the possible association of serum NGF and CGRP with osteoporosis and fracture need to be further investigated.

Altogether our data demonstrate that OVX and subsequent bone loss in mice do not induce pain-like behaviours and do not cause major changes in pain gene expression in bone and DRGs. However, OVX performed before an osteotomy showed worse outcomes in pain-like behaviours compared to an osteotomy performed in Sham OVX mice. These effects were not correlated with changes in pain gene expression in bone and DRGs. BDNF, CGRP and NGF were detectable in serum from patients with vertebral fractures and pain but our study was unable to demonstrate direct correlation between the serum concentrations of these markers and fracture pain reported by the patients. Nevertheless, our data show age and sex-related changes in serum protein levels of these markers that will need to be taken into account in the future studies.

## Supplementary Information

Below is the link to the electronic supplementary material.

Supplementary file1 (DOCX 297 kb)

## Data Availability

All data are available at the request of the authors.
