# Supplementary material for: Pain after osteoporotic fractures using mouse models and patient samples
Source: J Bone Miner Metab. 2025 Dec 25;44(1):132–46. doi: 10.1007/s00774-025-01677-w (PMC12891006; doi:10.1007/s00774-025-01677-w)
Supplement: Supplementary file 1 — Supplementary file1 (DOCX 297 kb) [file 774_2025_1677_MOESM1_ESM.docx]

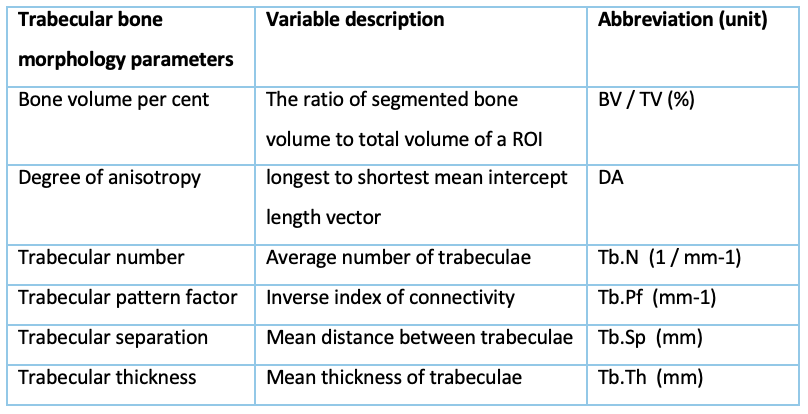

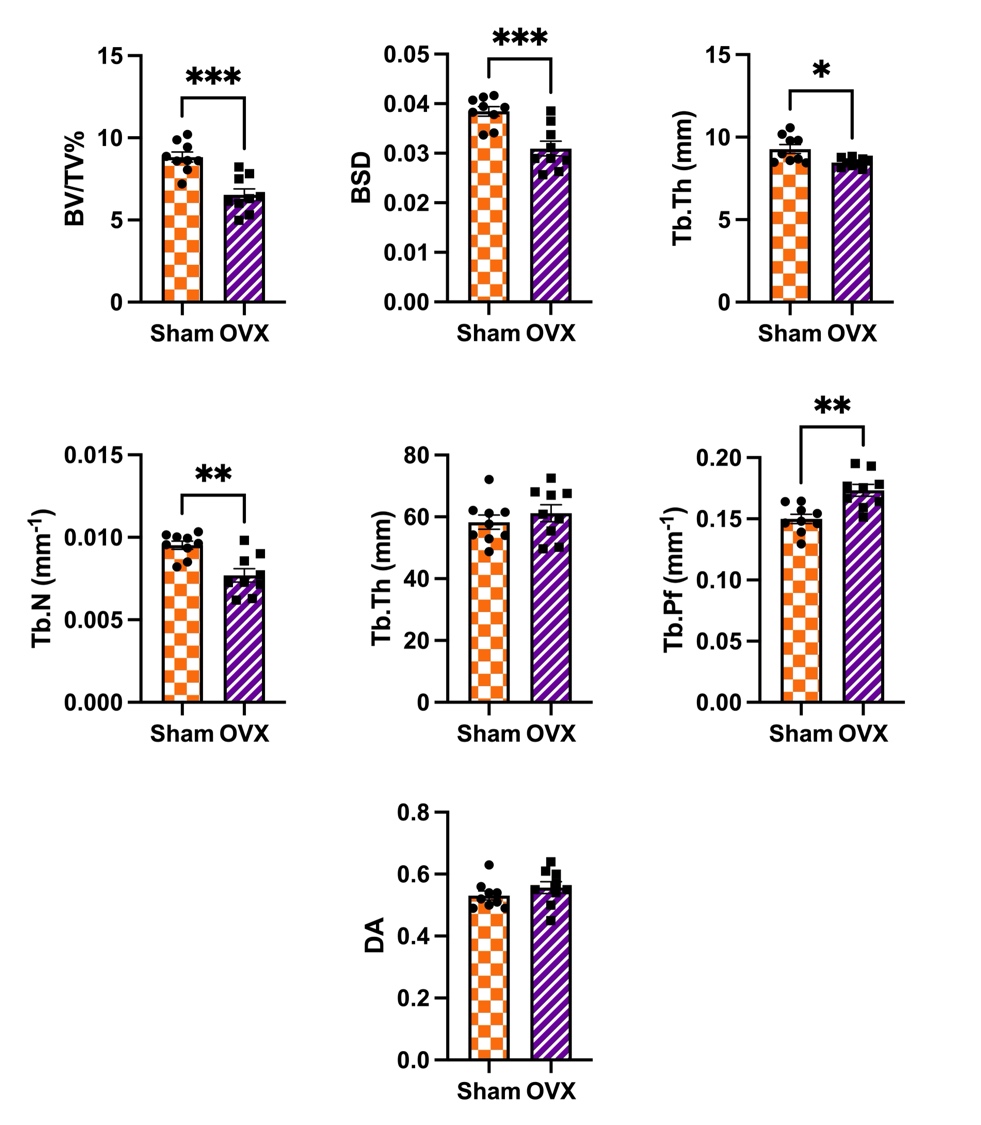


**Supplementary Figure 1**

**Micro-CT analysis of trabecular bone in OVX vs Sham mice revealing a loss of trabecular volume after ovariectomy.** 10-12 Week old C57BL/6 female mice were ovariectomised (n=10) or Sham operated (n=10). CT analysis of the trabecular bone in tibia has been performed 6 weeks after OVX. Results show M ± SEM and are normally distributed. For differences between OVX and Sham, sample-t tests have been performed and significance is shown as * (p-value<0.05) **(p-value<0.01) ***(p-value<0.001). Analysed parameters explained in adjacent table.
